# COVID-19 Influenced Survey on Students' Satisfaction With Psychological Acceptance Based on the Organization of Online Teaching and Learning in English

**DOI:** 10.3389/fpsyg.2022.940527

**Published:** 2022-07-13

**Authors:** Hua Zhang, Xinguo Li

**Affiliations:** ^1^School of Foreign Languages, Weifang University, Weifang, China; ^2^School of Foreign Languages, Anhui Polytechnic University, Wuhu, China

**Keywords:** online teaching, psychological disorder, numerical analysis method, satisfaction scale, satisfaction survey

## Abstract

Due to the epidemic, many offline educational institutions and even schools have begun to seek new teaching methods, and online teaching has become an important teaching method in this situation. Online teaching refers to the teaching mode in which teachers teach online through the Internet and students learn in online classrooms. Online teaching can reduce crowd contact between students and prevent the spread of the epidemic. However, due to the virtual nature of the Internet and the immaturity of the online teaching system, the current online teaching method cannot be accepted by the majority of teachers and students. Therefore, this paper conducts a satisfaction survey on the psychological acceptance ability of students under the online teaching organization of English majors, aiming to understand the actual psychological burden brought by online teaching to students. The content of the questionnaire includes students' satisfaction with courses and teaching, satisfaction with teacher-student interaction, satisfaction with platforms and resources, satisfaction with teaching performance, and overall satisfaction. The survey results show that students' satisfaction with courses is generally above 4 points, while their satisfaction with teaching is generally lower, ranging from 3.65 to 3.94 points. Students' satisfaction with teacher-student interaction is not very high, around 3.75. Students' satisfaction with the teaching platform is higher than that of teachers, with a score of 4.05 or more. In terms of students' satisfaction with teaching performance, male students' satisfaction is above 4.45 points, while female students' satisfaction is 4.35 points. For overall satisfaction, male students scored above 4.45, while female students scored 4.35. Since the satisfaction in all aspects is higher than 3 points, it can be seen that most English majors are quite satisfied with the online teaching.

## Introduction

Higher education systems around the world are facing challenges from new information and communication technologies due to the impact of the pandemic. These technologies have had a huge impact on the world economy, business management and globalization trends. They have enormous potential to reshape the nature of learning environments around the world. The Internet provides opportunities to acquire cross-cultural and individualized knowledge for learning, to acquire theoretical knowledge, and to explore and apply knowledge. The Internet provides globally accessible knowledge and learning applications anytime, anywhere. One of the learning applications that has become widespread is online learning. Existing and emerging e-learning technologies are having a strong, immediate and disruptive impact on the education system. Nowhere is this impact more felt than in teaching practitioners. More specifically, education has entered its third decade of profound changes in the way courses and programs are designed and delivered. During this time, many new possibilities became apparent. At the same time, with the rise of e-learning technology, many changes have taken place in almost all education sectors. Online teaching can be defined as the acquisition of knowledge and skills through learning applications written, communicated, supported and managed using Internet technologies. It is often referred to as e-learning. Online learning has become one of the most beneficial applications in higher education. Online courses and programs continue to grow in higher education institutions. Students are increasingly demanding internet access, so universities and colleges are improving their systems to meet their needs. Online teaching is an instructional delivery system that allows students to participate in an educational space without physically being in the same location as the teacher.

The economic downturn of the Great Depression has increased demand for college degrees, which can improve an individual's prospects for employment in a competitive, globalized marketplace. Social science research shows that students with low SES have less chance of succeeding in a university setting than students with high socioeconomic status (SES). Therefore, these students need to learn more knowledge through online learning in order to better seek jobs.

For English teaching, knowledge of letter pronunciation is a strong predictor of students' ability to decode words. About half of the English words can be decoded just by following the sound-symbol correspondence rules. Many students who are dyslexic or have difficulty learning to read have phonological difficulties understanding how sounds are organized in language. This can lead to difficulties in mastering the principles of letters, the relationship between sounds, and the letter patterns that represent them.

In order to investigate the psychological receptivity of English majors to online teaching, this paper uses the methods of numerical analysis and questionnaire survey to investigate the students' satisfaction. By investigating the satisfaction of 102 female college students, 18 male college students and 30 English teachers on all aspects of online teaching, the results show that students are more satisfied with the courses than teachers, and slightly less satisfied with the interaction between teachers and students. Because the enthusiasm of students is not high in the case of online teaching. Although teachers' Q&A is more active, due to the problems of the Internet, answering questions cannot be as smooth as face-to-face, so the overall satisfaction is not very high. Students are more satisfied with the teaching platform than teachers. The satisfaction of male students with teaching performance has decreased from very satisfied at the beginning, and finally showed an upward trend, while female students have been relatively satisfied. In terms of overall satisfaction, except for freshmen, male students in sophomores and juniors are much more satisfied than female students. Sophomore male students scored 4.87 points, junior male students scored 4.6 points, while sophomore female students scored only 4.3 points and junior female students only scored 4.32 points. But the overall result is still quite satisfactory.

Due to the impact of the epidemic, the previous offline teaching methods can no longer meet the current environment. The innovation of this paper is that it proposes a method of combining numerical analysis and questionnaire survey to investigate the satisfaction of students' psychological acceptance ability of English majors in online teaching. And combined with the calculation of reliability and validity, the results obtained have certain reliability.

## Related Work

Due to the impact of the epidemic, the traditional offline English teaching method can no longer meet the current environment, and many scholars have studied the teaching method of online teaching. Among them: Research by Asghar shows that the rapid growth in the number of distance education courses and programs has had a profound impact on perceptions and beliefs about teaching and learning (Asghar, 2020). Compared with ordinary teaching software, the network-based learning platform is more complex, which is reflected in the design thinking and technology application. Wang L improved the system design and development of the online teaching platform based on asp.net technology. Through the functional test of each functional module, it is proved that the basic functions of the designed online teaching platform can be realized. In order to meet the needs of general education informatization, the design of the operation interface is more user-friendly, allowing ordinary users to easily complete the operation of various functions. Wang L's research can help teachers improve teaching efficiency through advanced teaching methods (Wang and Wang, 2017). Adnan M provided insights into multinational teacher development programs for online teaching, detailing the results of an expectation and satisfaction survey. He collected data using an e-learning readiness and expectations questionnaire and used open-ended questions to measure satisfaction after the program. Descriptive statistics for analysis of survey data and content analysis of open data (Adnan et al., 2017). Based on post-positivist theoretical research, Glass explored the characteristics of experiences that shape attitudes toward online education among 16 faculty members. Two characteristics were identified: their efforts to express topics of personal significance, and their efforts to assume a variety of social roles. This analysis focuses on recognizing that online education has not only changed the way faculty members teach, but also the way teachers teach (Glass, 2017). Although these online teaching methods can be applied to the epidemic environment, they will also have some psychological effects on students.

In order to understand the impact of online teaching on students, some scholars have conducted investigations from various perspectives. Jury M reviewed the psychological barriers faced by low SES students compared to high SES students in higher education. And he presented three examples of psychological interventions that can potentially improve academic performance and the quality of the experience of low-SES students, and thus can be seen as approaches to change (Jury et al., 2017). Gentry research has shown that intensive, explicit instruction is beneficial for developing struggling readers' ability to recognize phonemes and apply knowledge of phoneme-grapheme correspondences for decoding. Gentry provided common misconceptions and basic principles for effective letter pronunciation instruction to help special educators and reading interventionists develop effective phoneme-grapheme correspondence instruction programs for students with dyslexia or at risk of reading failure (Earle and Sayeski, 2017). Ali suggested that medical education research should address the needs of international healthcare students and find strategies to meet their needs in classroom and clinical learning settings (Anoushirvani and Golaghaie, 2019). Markey M focused on how bilingual students can leverage previous language experience when learning a new language. In addition to measures of proficiency, the findings also point to the need for teaching tools that allow students to make the most of their previous language experience (Markey, 2022). These survey methods are relatively traditional, inefficient, and have individual subjective factors, which are dependent on the sample, and their credibility needs to be verified.

## Numerical Deconstruction Satisfaction Survey of Online Teaching

### Online Teaching

The online autonomous English learning model is based on the actual situation of English teaching in China, and is based on constructivism and dual subject theory. Based on the network multimedia technology environment, to play the main role of teachers, to provide a learner-centered stage, so that teachers and students can discover their respective roles, Transforming feelings and thirst for knowledge unconsciously into a positive and active state, teachers and students jointly explore a learning path that conforms to the characteristics of learners, and jointly create a network-based autonomous learning environment for teaching and learning (Rhode et al., 2017).

An increase in the number of international students in the higher education system is seen as beneficial not only financially, but also in terms of preparing the workforce for a global environment. It is reported that the diversity of the student body can also benefit domestic students in increasing cultural awareness and achieving cultural competency goals. Therefore, the educational issues of college students need to be researched and addressed to increase their motivation for academic success. The online education based on network teaching is a good method (Javid et al., 2021; Liu H. et al., 2021).

Online teaching requires different skills, roles and competencies of online teachers compared to teaching in traditional learning environments. In the context of the pandemic, universities should provide ongoing support in various forms to assist academic staff in their online journeys. There is currently a growing gap between managers' and faculty's attitudes toward online education, as online education has always been controversial because of the virtual nature of the network (Baber, 2020; Gupta and Chaudhary, 2020; Zaher et al., 2020; Fang and Li, 2021).

The actual situation of basic education is that most of the learning activities take place in the classroom, and the classroom is the main environment for English learning. Therefore, teachers pay more attention to how to use the Internet in the classroom environment to create a more effective learning environment suitable for learners. In the actual online teaching, it is necessary to use the network to supplement teaching resources. In the course of preparing lessons, the network is used to search for relevant materials to supplement and expand teaching resources. This method provides students with rich and vivid learning materials, but in essence, the Internet is still an auxiliary tool for teachers and does not give full play to the role of assisting students in learning. Design teaching web pages. According to the unit teaching system, guide students to study online. Practice has found that this model of classroom teaching regulation is very difficult, and the biggest problem is that it is time-consuming and labor-intensive, with high professional and technical requirements, which cannot be completed by teachers alone. Display multimedia equipment. Practice has proved that this is a simple, easy and effective way to give full play to the resource advantages of the network. It is also the most frequently used method in teaching practice in the epidemic environment (Alli, 2021; Bulut and Del, 2021).

Teachers need a good network environment and hardware equipment in the process of network teaching. However, due to the phenomenon that some students do not have computers, or the students dormitories do not have Internet access, etc., the quality of teaching declines. This is a reality that is difficult to change in the short term. Under the existing conditions, how to use the autonomous learning center more fully has become the main problem of using the network teaching system to assist students' autonomous learning. At the same time, in the learning of online courses, the communication between teachers and students cannot be as direct and convenient as traditional classroom teaching, and it is difficult for students to effectively enter the learning state, and it is difficult to maintain long-term attention. Compared with classroom teaching, it is not easy to keep students' attention and focus on learning material for a long time. These are all new challenges that online education presents to teachers and students. Solving these problems is a prerequisite for promoting English online self-learning (Fitriani et al., 2021; Liu P. et al., 2021). In addition, the model of China's online education is still only in the “hot” stage. Simply put the curriculum and teaching materials in the offline learning mode on the Internet, and conduct one-way cramming teaching for all learners without any difference.

The advantage of online teaching is that it can study anywhere, and there are many kinds of online courses. Students can arrange their favorite courses to study according to their own learning situation. In addition, the learning of the course can be watched repeatedly to check and fill in the gaps. Compared with offline teaching, the learning cost is much lower. However, some schools currently use online teaching for up to a year, and there is no corresponding training activities for foreign language teachers to teach online. It also does not use the network testing function and its derived grading function of the network itself. The network management personnel of the Academic Affairs Office do not maintain the “New Concept” in their daily work, the school does not have a special person responsible for the management of the “New Concept,” and the network failure cannot be dealt with in time. There is no monitoring of students' learning quality, and no assessment indicators for teachers' online teaching quality. This leads to the lack of detailed plans for English online teaching; the unclear division of functions between the equipment department, the academic affairs office and the foreign language college; and the lack of assessment indicators for the quality of teachers' teaching and students' learning. How to improve the teaching management of online autonomous learning has become the guarantee for the smooth development of English online autonomous learning (Sato and Chen, 2021; Ullah et al., 2021).

Due to the virtual nature of the network, there are many defects in online teaching. Among them, the outdated concept of teacher education is also a problem faced by online teaching. For example, the number and frequency of teachers logging in, leaving messages, answering student questions, and uploading materials can reflect many foreign language teachers believe that only classroom teaching can achieve English learning goals, and they are still keen on classroom face-to-face teaching. They have doubts about the use of college English online learning platforms, and ignore the responsibilities that teachers should assume in online self-learning. Therefore, how to make teachers with an open mind, actively adapt to changes, improve teachers' awareness of online self-learning, and change teachers' negative views on online learning are problems that need to be solved. For college students, they already have considerable autonomous learning ability, and various forms of learning materials are very rich. Computer networks and various learning tools provide great convenience for college students to conduct English autonomous learning. What students need is knowledge and help that they cannot get as individual learners. Such as testing the learning effect, face-to-face English communication, solving difficult problems, how to formulate study plans, how to choose learning materials suitable for individuals, how to effectively use the Internet for learning and so on. The responsibility of teachers is to solve the above problems that cannot be solved by individual students (Khtere and Yousef, 2021; Zhang et al., 2021).

In addition to teachers' problems with online teaching, there are also problems students face with online learning. The limitations brought about by online teaching can lead to students learning freely and loosely, resulting in anxiety, lack of patience, and even frequent concerns about their own grades when they cannot keep up with their learning progress. Due to the lack of supervision of students' learning in the online classroom, some students even open several windows, log in to the online classroom, play computer games, and practice typing. And students are not yet proficient in using various information tools. This shows that students lack the ability of information immunity, information cooperation and the ability to use information tools. And information literacy is a prerequisite for students to learn independently online. At present, students' self-learning knowledge awareness is generally low. Strategic learning knowledge is the basic learning behavior guarantee for students to carry out English online self-learning, and it determines the efficiency of English online self-learning. Therefore, it is necessary to improve students' information literacy and autonomous learning ability.

### Numerical Deconstruction

When conducting a satisfaction survey, the first thing to do is to test the reliability of the survey content. If T is the actual value, B is the deviation, E is the other error, and X is the test value, then:


(1) $$\begin{array}{l} X=T+B+E \end{array}$$

Since B can be minimized in the design process of the questionnaire and can be ignored in the reliability test, then:


(2) $$\begin{array}{l} X=T+E \end{array}$$

For other errors E, let its expectation be 0, then:


(3) $$\begin{array}{l} E\left(X\right)=E\left(T\right) \end{array}$$

In addition, the actual values T and E are independent values, so:


(4) $$\begin{array}{l} Var\left(X\right)=Var\left(T\right)+Var\left(E\right) \end{array}$$

Among them: Var represents the variance of the test value.

The reliability of the questionnaire is denoted by R and can be defined as:


(5) $$\begin{array}{l} R=\frac{Var\left(T\right)}{Var\left(X\right)}=1-\frac{Var\left(E\right)}{Var\left(X\right)} \end{array}$$

Or:


(6) $$\begin{array}{l} R=\sqrt{\frac{Var\left(T\right)}{Var\left(X\right)}} \end{array}$$

When the R value is smaller, the reliability of the questionnaire is smaller, and vice versa.

In addition to the reliability test, it is also necessary to test the validity to judge the validity of the results. For the CVI validity (That is, content validity index refers to the degree of agreement between the content actually measured by a scale and the content to be measured, and content validity is an important reflection of the quality of the scale.), it is:


(7) $$\begin{array}{l} CVI=\frac{A}{n} \end{array}$$

Among them, A represents the number of content identical results, and n represents the number of content items.

And the validity for all content can be calculated as:


(8) $$\begin{array}{l} Pc=\left[\frac{n!}{A!\left(n-A\right)!}\right]*0.5^{n} \end{array}$$

Among them,


(9) $$\begin{array}{l} n=\frac{z^{2}\sigma^{2}}{d^{2}} \end{array}$$

For the content validity ratio, it can be calculated as:


(10) $$\begin{array}{l} CVR=\frac{\left(n_{e}-N/2\right)}{N/2} \end{array}$$

Among them: N is the number of ratings, and *n*_*e*_ is the number of relevant content.

The satisfaction calculation method for the data is:


(11) $$\begin{array}{lll} Si & = & \frac{A+O}{A+O+M+I}*1 \end{array}$$

(12) $$\begin{array}{lll} Dsi & = & \frac{O+M}{A+O+M+I}*\left(-1\right) \end{array}$$

The weights for the indicators of the survey items can be calculated as:


(13) $$\begin{array}{l} y_{ij}=\frac{x_{ij}}{\sum_{i=1}^{m}x_{ij}} \end{array}$$

The information entropy value for the indicator is:


(14) $$\begin{array}{l} e_{j}=-\frac{\sum_{i=1}^{m}y_{ij}in_{y_{ij}}}{\ln_{m}} \end{array}$$

Combined with expert scores to get weights:


(15) $$\begin{array}{l} w_{j}=\frac{{\left(1+d_{j}\right)}^{*}H_{j}}{\sum_{j=1}^{n}\left(1-e_{j}\right)} \end{array}$$

The weight assignment obtained by Formula (15) can finally obtain the satisfaction:


(16) $$\begin{array}{lll} Si & = & \frac{W_{1}A+W_{2}O}{W_{1}A+W_{2}O+W_{3}M+W_{4}I}*1 \end{array}$$

(17) $$\begin{array}{lll} DSi & = & \frac{W_{2}O+W_{3}M}{W_{1}A+W_{2}O+W_{3}M+W_{4}I}*\left(-1\right) \end{array}$$

In addition, when prioritizing the evaluation results, the sorting method is as follows:


(18) $$\begin{array}{l} D_{i}=\overset{n}{\underset{j=1}{\sum }}\frac{W_{J}|X_{0}-K_{ij}|}{\left|M_{j}\right|} \end{array}$$

## Design and Deconstruction of Satisfaction Questionnaire

This research will carry out a survey of college students, and use quantitative data to describe the satisfaction of college students with English online teaching. Quantitative surveys need special survey tools to ensure the validity and reliability of survey results through survey tools with certain validity and reliability. As shown in the results of the review in Chapter 2, there is no measurement tool specifically used to investigate the satisfaction of college students in English online teaching in previous studies. Therefore, the primary research content of college students' English online teaching satisfaction survey research is the development of measurement tools. This chapter will introduce the process and results of the research on the satisfaction scale of college students' English online teaching.

### Design of the Satisfaction Scale for College Students' English Online Teaching

#### Determination of Scale Dimensions

Satisfaction research originated from the research on customer satisfaction in the field of marketing. In the field of education research, there are also surveys about the satisfaction of college students with the quality of education or course quality, such as the “British Student Satisfaction Survey” (NSS), “Australian Curriculum Experience Questionnaire” (CEQ) and so on. Previous studies have shown that student satisfaction is a complex and ambiguous concept that includes multiple dimensions. To investigate the satisfaction of college students in English online teaching in this study, it is necessary to initially determine the specific dimensions of English online teaching satisfaction. The methods of determining the dimensions include literature research and expert consultation.

By sorting out relevant literature and understanding the actual situation of college students' English online teaching learning, this study initially determined six dimensions, which are course content, teachers' teaching, students' learning, teacher-student interaction, evaluation, and platform support. The meaning and content of each dimension are defined as follows. Dimension 1: Course content. Specifically, it refers to the knowledge, skills, concepts, tasks or activities presented to students by the course, and it answers the question of “what to teach and what to learn.” Dimension 2: Teacher's teaching. Specifically, it refers to the explanation, demonstration and guidance of teachers on knowledge, skills, concepts, tasks, etc. in the teaching process. Dimension 3: Learner learning. Specifically, it refers to the attitude, motivation, engagement and behavior of learners in the learning process. Dimension 4: Interaction. Specifically, it refers to the interaction between teachers and students and between students and students in the process of English online teaching. Dimension 5: Evaluation. Specifically, it refers to the assignments, tests, examinations, etc. that students are required to complete, which are used to consolidate, test, and evaluate students' learning. Dimension 6: Platform support. Specifically, it refers to the learning environment and support provided by online teaching in English, including learning resources, retrieval functions and other services.

The rationality of the dimensions of the scale determines the content validity of the scale, that is, the consistency between what the scale actually measures and what it expects to measure. After the dimensions of the scale are preliminarily formulated through literature review, in order to improve its content validity, it is necessary to invite experts other than the scale compiler to judge it. Most of the experts consulted in this research are teaching or research staff of curriculum and teaching theory or educational technology, and they all have doctoral degrees or senior professional titles. Advice was collected through a written questionnaire. Each dimension and its meaning are presented in the questionnaire, and then experts are asked to judge whether this dimension is an important aspect of college students' satisfaction with online teaching in English, and make the only choice among the three options.

#### Deconstruction of Scale Reliability

The reliability analysis here aims to examine the degree of internal consistency of the scale. In this study, the Cronbach's value was used to test the consistency of participants' responses to each dimension (i.e., factor) as well as the entire scale. As shown in Table 1, the Cronbach value of each dimension is above 0.8, and the Cronbach value of the entire scale is 0.965. The above results show that the scale has good reliability.

**Table 1:** Satisfaction scale values.

**Index**	**Name**	**Number of questions**	**Coefficient value**
I1	Satisfaction with curriculum and teaching	5	0.879
I2	Satisfaction with platform and resources	3	0.896
I3	Satisfaction of teacher-student interaction	4	0.943
I4	Satisfaction with teaching performance	3	0.627
Overall		15	0.965

### Design and Deconstruction of the Questionnaire

#### Determination of the Object of Investigation

Due to the limitation of research conditions, this survey only takes undergraduates and teachers of English majors in a certain university as the survey objects, so as to make a preliminary exploration on the actual situation of college students' satisfaction with English online teaching. Its questionnaires including satisfaction scales were made into online questionnaires. Questionnaire links are generated through the Questionnaire Star platform for college students taking courses to read and voluntarily participate in the survey. In the end, a total of 150 questionnaires were collected, including 120 questionnaires for college students and 30 questionnaires for teachers. The basic information of the students surveyed is shown in Table 2, and the basic information of the teachers surveyed is shown in Table 3. From the data in the table, it can be seen that the survey objects are mainly college students, mainly third-year undergraduates. Among the survey respondents, boys account for 12% of the total sample, and girls account for 88%. The number of girls far exceeds that of boys, which has a certain relationship with the selected English major (For girls, many science and engineering majors are not only difficult to study during the undergraduate period, but also very difficult to find employment after graduation. Therefore, in order to choose a major with relatively easy, stable and better salary after graduation, they will have less employment pressure in the future. Then the English major has become the first choice for most girls to apply for the major, so there are more girls majoring in English).

**Table 2:** Basic information of the surveyed college students.

**Attributes**	**Number of samples**	
Gender	Male	18
	Female	102
Educational level	Undergraduate	120
Grade	Freshman	12
	Sophomore	15
	Junior	93

**Table 3:** Basic information of teachers surveyed.

**Attributes**	**Number of samples**	
Gender	Male	19
	Female	11
Educational level	Undergraduate	15
	Master	10
	PhD	5

#### Determination of the Content of the Investigation

The overall satisfaction is to judge the satisfaction of college students with English online teaching in each dimension by calculating the mean value of each dimension. Since the satisfaction scale adopts a 5-point scoring system, from high to low corresponds to very satisfied and very dissatisfied, so 5 is the highest satisfaction, 1 is the lowest satisfaction, and 3 is the middle value. When the average value is higher than 3 points, it means that college students tend to be satisfied with English online teaching, and if it is lower than 3 points, it means that college students tend to be dissatisfied with English online teaching.

The content of the survey of teachers and students' satisfaction with the curriculum and teaching is shown in Table 4. It is mainly divided into the survey of curriculum support dimension and the investigation of teacher teaching support dimension.

**Table 4:** Survey content of teachers and students' satisfaction with curriculum and teaching.

**Subdimension**	**Specific contents**	**Weight**
Subdimension:	The difficulty of this course is acceptable to me	0.35
Course	The content of this course can stimulate the interest in learning	0.3
	The overall time schedule for this course is very reasonable	0.35
Subdimensions: Teachers'Teaching	The teacher's explanation of the knowledge points in the teaching process is clear and easy to understand	0.4
	Teachers can mobilize the enthusiasm for learning in the teaching process	0.25
	The teacher's teaching can link theory with practice	0.35

Satisfaction of teacher-student interaction, which includes the timely response of teachers to students' questions in the discussion area, and the ability of teachers to answer questions in the discussion area to solve students' questions, the teacher's participation in the classroom discussion area aroused the enthusiasm of the students to participate in the discussion, and the teacher gave serious responses to the students' questions or speeches in the discussion area. Most of the opinions expressed by online students in the classroom discussion area are in-depth, the discussion on the teaching content in the classroom discussion area can help students consolidate their knowledge, and students actively participate in the discussion because of their interest in the problem.

The satisfaction of teachers and students with the English online teaching platform and resources is based on the clear and beautiful interface of the course, the various forms of course resources, and the clarity of the video figure of the course meets the visual requirements of teachers and students, and the course reminder function of the platform has played a role in supervising students' learning.

The satisfaction of different subjects on teaching performance is based on the amount of homework students enter into the course, students' consolidating learning effect, the difficulty of test questions, and the self-evaluation of final grades.

### Deconstruction of Satisfaction Survey Results

**Figure 1:**
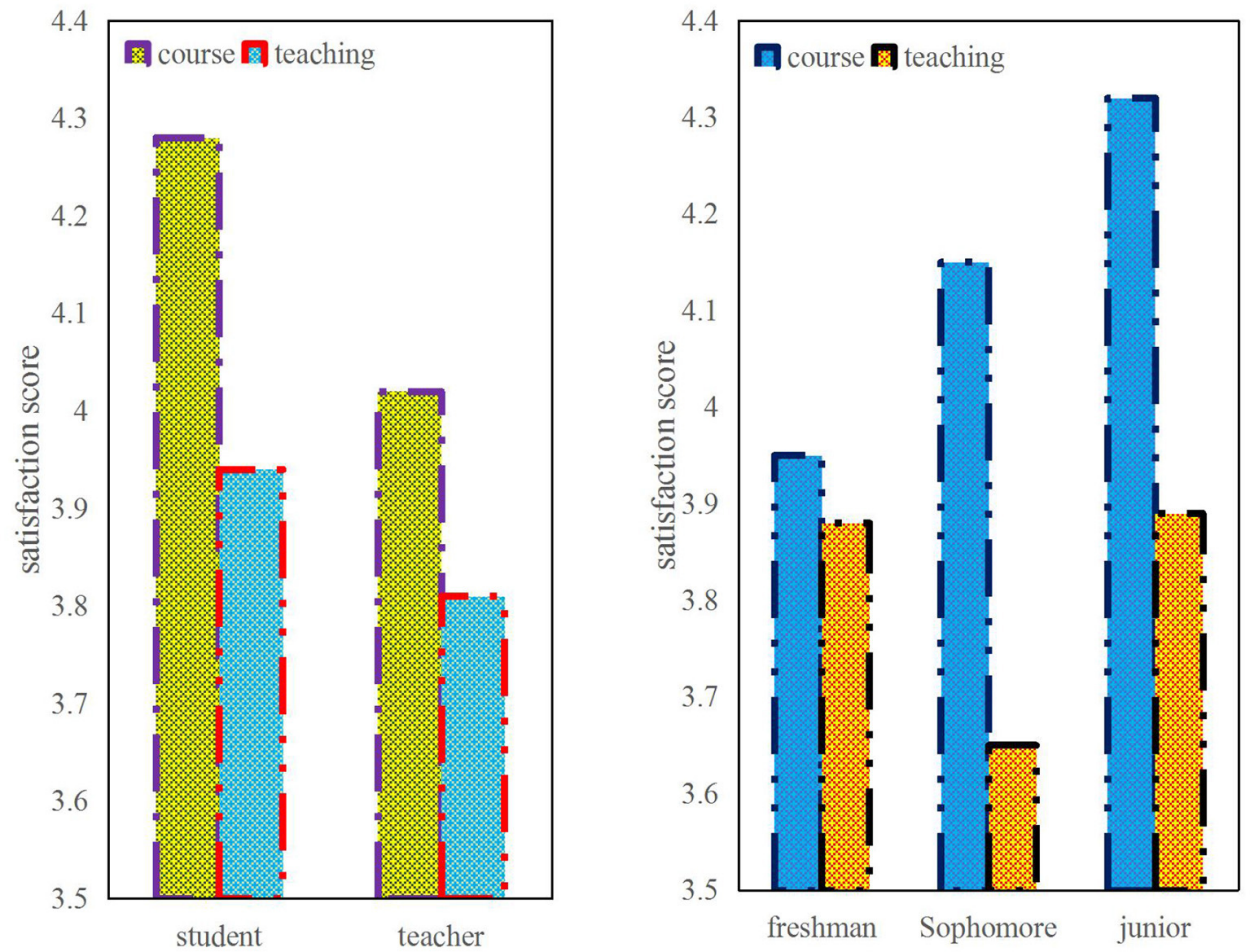
Comparison of different subjects' satisfaction with curriculum and teaching.

Figure 1 shows the satisfaction with the curriculum and teaching between the first and third year students and between teachers and students. It can be seen from the figure that the students' satisfaction with the course is higher than that of the teachers, but they are generally above 4 points, while the satisfaction with the teaching is generally lower, ranging from 3.65 to 3.94. It shows that the arrangement of online courses is more reasonable, but the teaching effect is not as good as expected. In addition, for freshmen, their satisfaction with courses and teaching is lower than that of juniors. Because freshmen do not feel the urgency of studying with courses, they are not as relaxed psychologically as juniors, so they are more disgusted with arranging course study.

**Figure 2:**
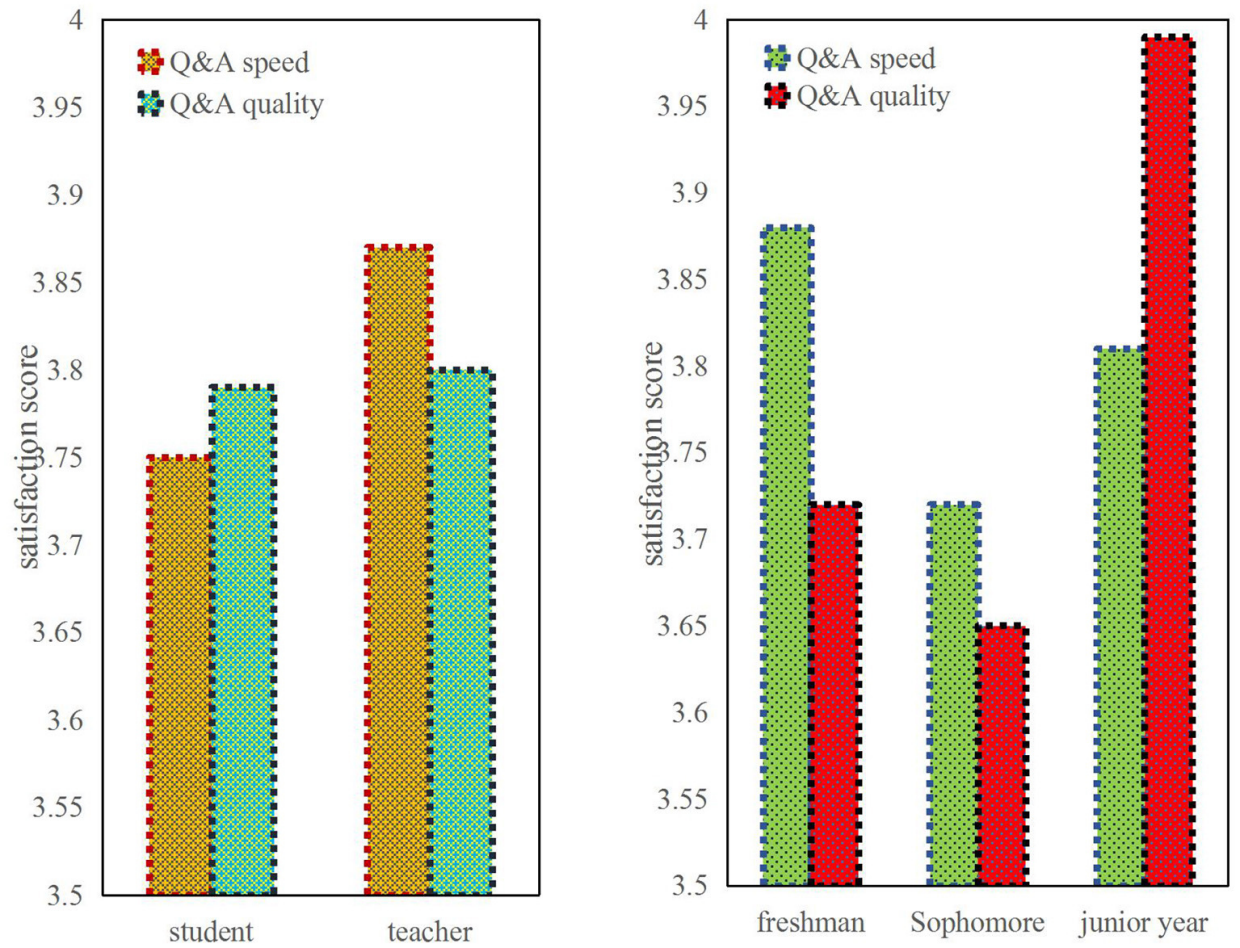
Comparison of satisfaction with teacher-student interaction among different subjects.

The satisfaction survey of teacher-student interaction during class can be seen from Figure 2. The students' questions and answers are not very satisfactory, around 3.75, while the teacher's answer is 3.88, which is relatively good. This shows that the enthusiasm of students is not high in the case of online teaching, although teachers' Q&A is more active. However, due to the problems of the Internet, answering questions cannot be as smooth as face-to-face. So overall satisfaction is not very high. And among the freshman to junior college students, it can be seen that the satisfaction of the freshman's question and answer is very high, because freshmen's questions may be relatively simple, teachers' answers are relatively quick. Therefore, the satisfaction of freshmen is higher, and the Q&A quality of juniors is significantly higher than that of freshmen and sophomores. Their satisfaction is high because they learn more and they can answer better questions asked by teachers.

**Figure 3:**
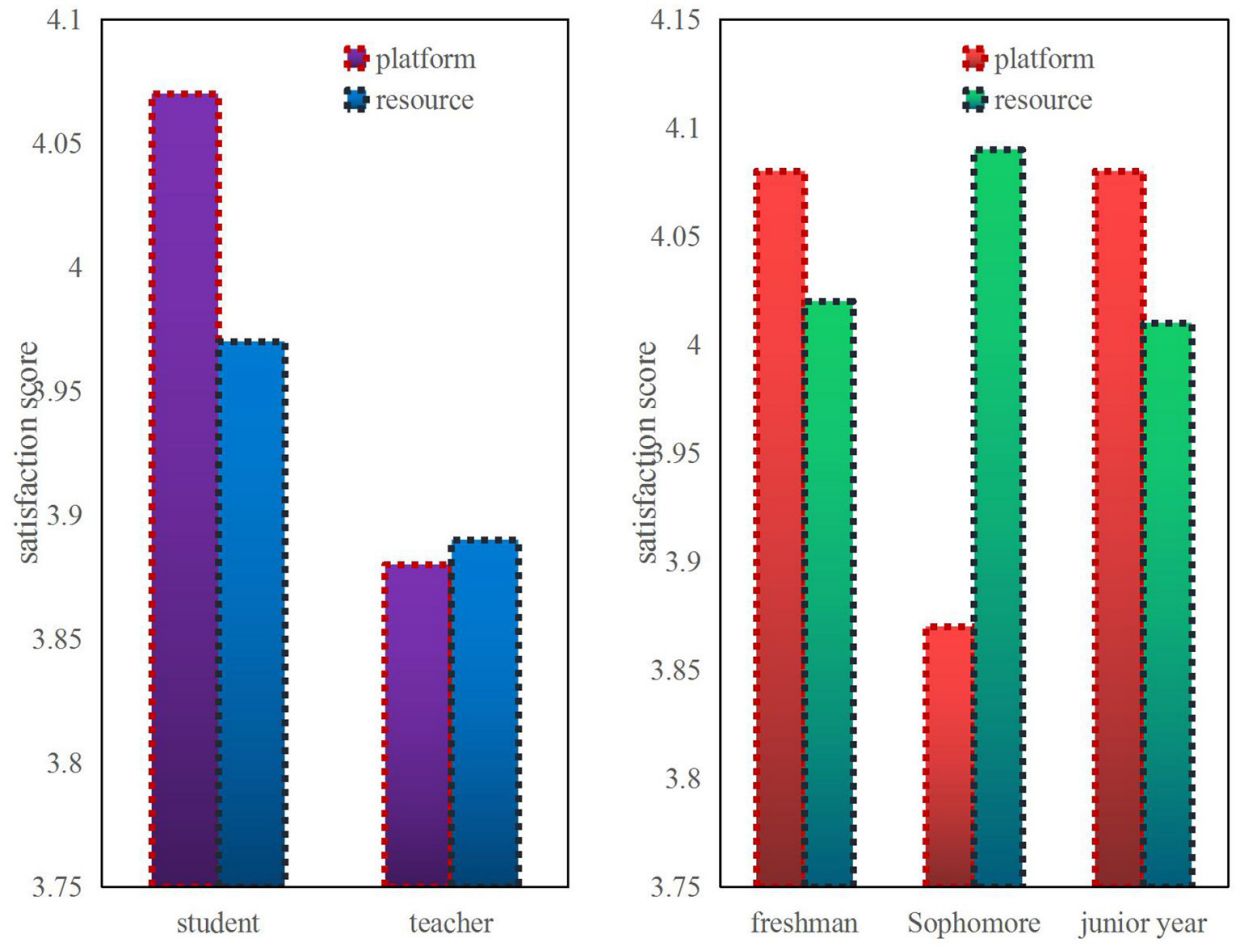
Comparison of satisfaction of different objects with platforms and resources.

The satisfaction of teachers and students with the teaching platform and teaching resources can be shown in Figure 3. Students' satisfaction with the teaching platform is higher than that of teachers, with a score of 4.05 or more. It may be that students feel that it is more convenient to conduct classes online, and that they can review the class content again after class. The teacher satisfaction is below 4.0, which may be because the teaching method of the teaching platform is not very suitable, and the use of teaching resources is not familiar.

**Figure 4:**
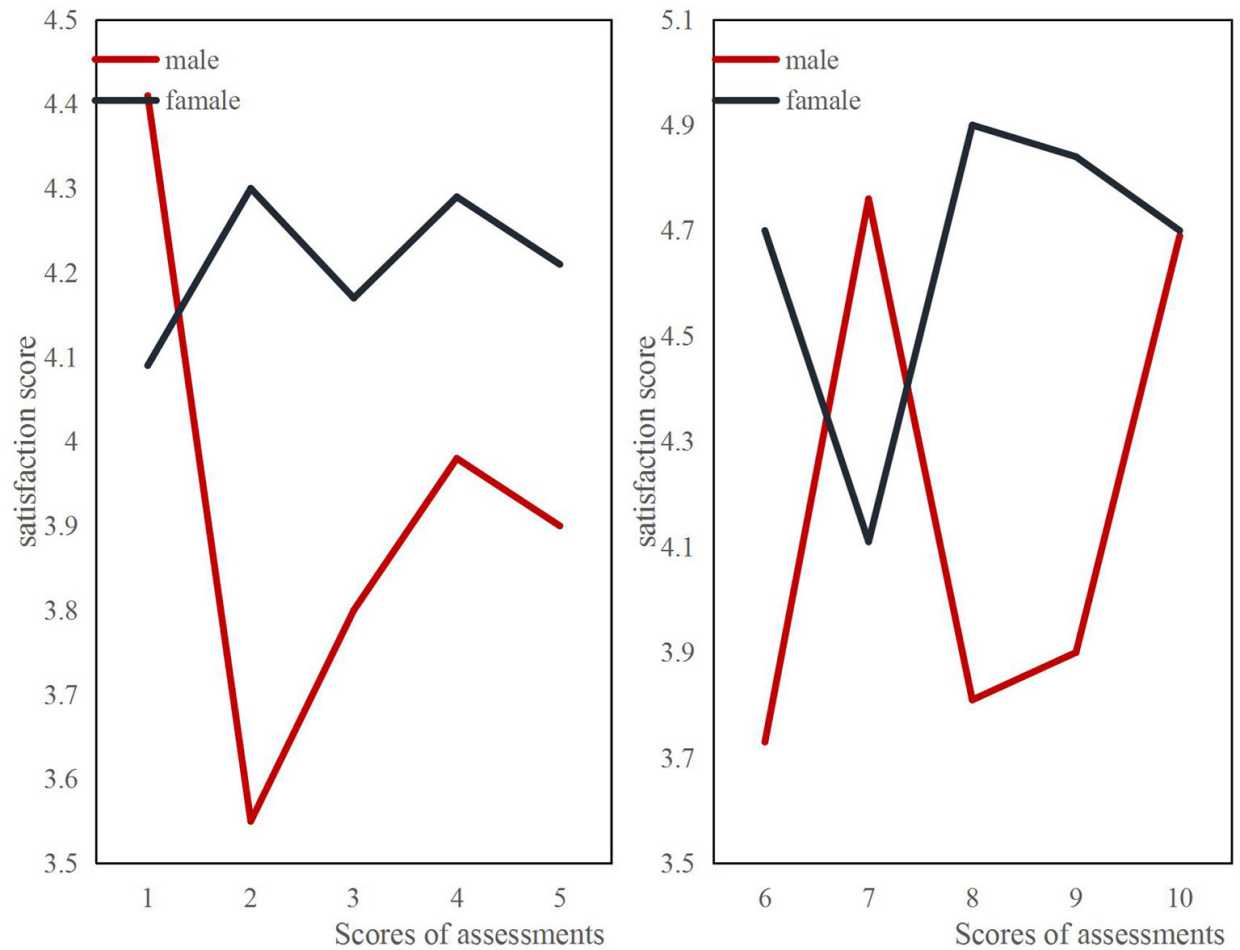
Comparison of male and female students' satisfaction with teaching performance.

The satisfaction survey of the students after the performance evaluation is shown in Figure 4. In the first 5 evaluations of male students, except for the first score evaluation, the satisfaction is higher, and the rest are lower than those of female students. It may be that male students are curious and interested in online teaching at the beginning, and their learning motivation is more powerful. But later, they gradually lose interest, their motivation to study also decline, and their grades naturally decline, resulting in a general decrease in male students' satisfaction with their teaching performance. In the next five grades assessments, because students are more able to adapt to this learning method, their grades will fluctuate up and down, but the overall trend is on the rise.

**Figure 5:**
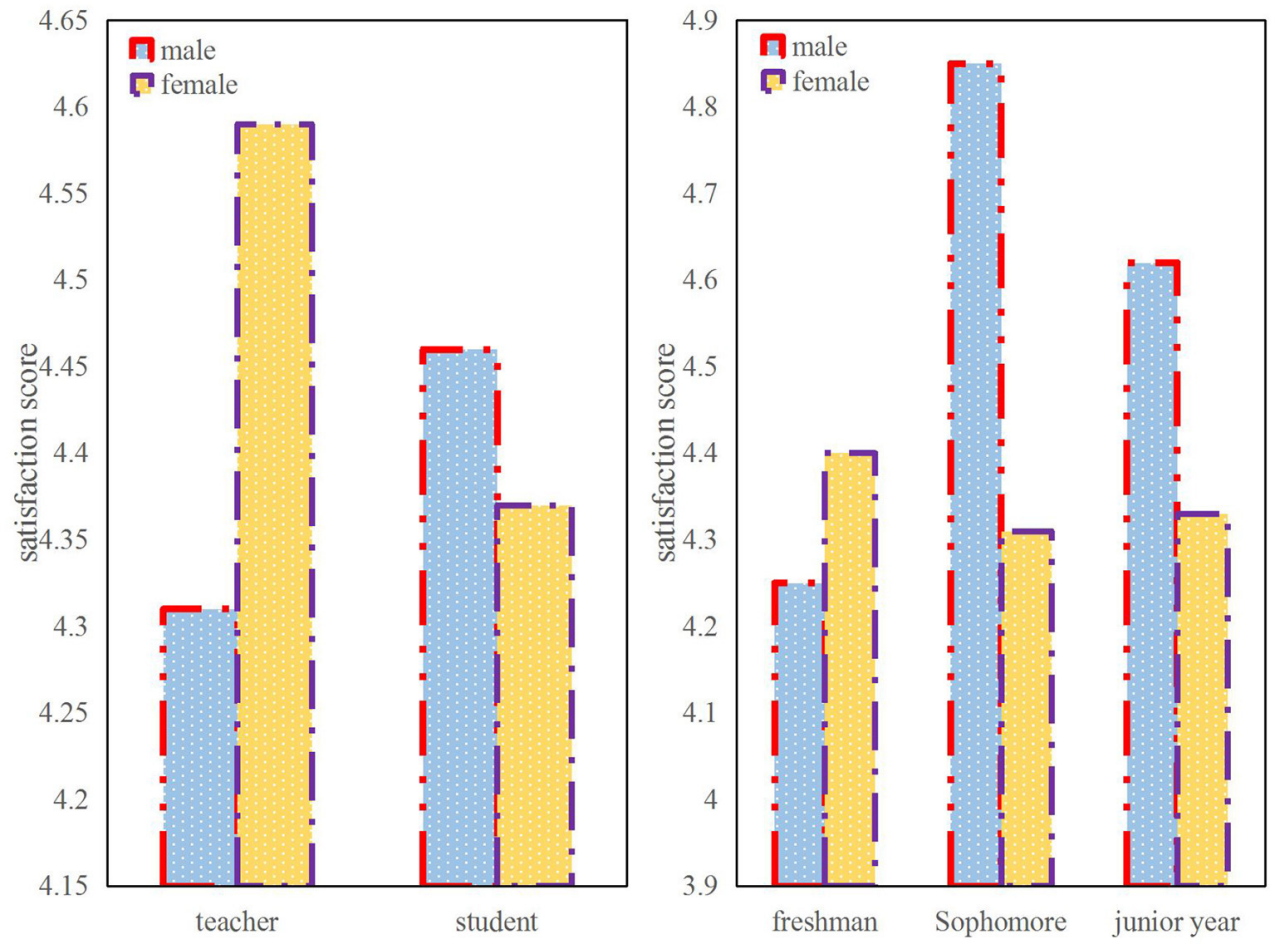
Comparison of overall satisfaction among different subjects.

The overall satisfaction survey is shown in Figure 5. Among them, the overall satisfaction of male teachers is relatively high, reaching about 4.6 points, while female teachers are relatively low, at 4.32 points. It may be because male teachers are more recognizable to this new education model and are more comfortable with the use of online teaching. Female teachers, on the other hand, are less recognized and more difficult to use online teaching. In addition, the overall satisfaction of male students is still relatively high, above 4.45 points, while that of female students is 4.35 points. In addition to the freshman year, the satisfaction of male students in the second and third year is much higher than that of the female students. Sophomore male students scored 4.87 points, junior male students scored 4.6 points, while sophomore female students scored only 4.3 points and junior female students only scored 4.32 points.

## Conclusions

This paper firstly summarizes the research purpose and content of this paper in the abstract section. In the introduction part, the defects of traditional education methods and the characteristics of online teaching under the background of the epidemic are introduced. The specific research methods and innovative points of this paper are introduced, and many examples related to the theme are listed in the related work to reflect the current situation of the research content of this paper. In the theoretical research part, this paper first focuses on online teaching, including its online teaching methods and requirements for online teaching. Then, the specific calculation method of numerical analysis method is introduced, including the calculation of reliability, validity and satisfaction. Finally, in the experimental part, the dimensions of the scale are first determined, and then a questionnaire is designed. According to the survey results, English majors are generally satisfied with their psychological acceptance under the online teaching organization.

As a result of the epidemic, previous offline teaching methods can no longer meet the current environment. The innovation of this paper is to propose a method combining numerical analysis and questionnaires to investigate the satisfaction of English majors' mental receptiveness in online teaching. It is also combined with the calculation of reliability and validity, and the results obtained have a certain degree of reliability.

## Data Availability Statement

The original contributions presented in the study are included in the article/supplementary material, further inquiries can be directed to the corresponding author/s.

## Author Contributions

HZ and XL were responsible for designing the framework of the entire manuscript from topic selection to solution to experimental verification. All authors contributed to the article and approved the submitted version.

## Funding

This work was supported by Anhui Provincial Education Quality Engineering Project under grant no. 2020jyxm0143, and Anhui Polytechnic University Research Project under grant no. Xjky2022207.

## Conflict of Interest

The authors declare that the research was conducted in the absence of any commercial or financial relationships that could be construed as a potential conflict of interest.

## Publisher's Note

All claims expressed in this article are solely those of the authors and do not necessarily represent those of their affiliated organizations, or those of the publisher, the editors and the reviewers. Any product that may be evaluated in this article, or claim that may be made by its manufacturer, is not guaranteed or endorsed by the publisher.
